# Self-Efficacy and Depression in Boxers: A Mediation Model

**DOI:** 10.3389/fpsyt.2020.00791

**Published:** 2020-09-29

**Authors:** Xin Chen, Nan Qiu, Chao Chen, Dexin Wang, Guodong Zhang, Liang Zhai

**Affiliations:** ^1^ Department of Sport and Health Sciences, Technical University of Munich, Munich, Germany; ^2^ General and Experimental Psychology, Department of Psychology, LMU Munich, Munich, Germany; ^3^ Shanghai University of Sport, Shanghai, China; ^4^ College of Physical Education, Institute of Sport Science, Southwest University, Chongqing, China; ^5^ College of Physical Education, Sichuan Agricultural University, Yaan, China

**Keywords:** self-efficacy, self-control, depression, mediating effect, boxers

## Abstract

**Background:**

Depression has become one of the most common problems faced by athletes. In many mental health problems, its production and development mechanisms and influencing factors have received full attention from researchers, whereas boxers’ depression has received limited attention. This study explored the relationship between boxers’ self-efficacy and depression, as well as the effect of self-control as a mediating factor.

**Methods:**

This study used the athlete self-efficacy scale (ASES), the self-control scale (SCS), and the Center for Epidemiologic Studies depression scale (CES-D). Using a large number of randomly selected samples, a total of *N*=231 boxers (age: *M* =20.28, *SD* = 2.60, ages around 18 to 32; the total number of years of exercise: *M* = 6.07 years, *SD* = 2.90, years around 1 to 15; 144 male) of Chinese national athletes participated the study.

**Results:**

Self-efficacy and self-control were negatively correlated with depression; self-efficacy was positively correlated with self-control. In addition, self-control played a partial mediation role between self-efficacy and depression among boxers.

**Conclusion:**

Above all, an important way to improve and prevent the depression of Chinese boxers maybe enhance their level of self-efficacy and self-control.

## Introduction

The mental health of elite athletes is increasingly becoming the focus of attention both in sports psychology and sports medicine (1–4). However, few studies have examined elite athletes’ coping strategies for mental health problems such as depression. Such research is vital: depression is a widespread social problem affecting both the general population and athletes (5–7). While the exact prevalence of depression among elite athletes is still unclear (2, 3) and often underestimated (8), the extent to which depressive symptoms occur among them is currently considered comparable (9, 10) or higher (11) to that among the general population. Previous studies have also found that athletes engaged in team sports have lower levels of depression than those who participate in individual sports (12).

Scholars in sport psychology have called for research that ‘gives a voice’ to marginalized groups (13, 14), which would arguably include boxers. Boxing is a combat sport that places two individuals in intense one-on-one physical and mental competition, who not only need to rapidly lose weight before the game to maintain the best competitive state, but also face the risk of concussion during competition and training. Research has also found that rapid weight loss and concussion are associated with depression among samples of boxers (15, 16). However, these findings which showed a link between depression and sport-related concussion, did not suggest any relationship between depression, self-control, and self-report methods among boxers.

Neurobiological and psychosocial models at this stage give evidence for the relationship between brain injury and depression. On the one hand, the neurobiological model of depression has provided compelling and parsimonious accounts of depression (17, 18). For example, individuals with clinical depression have been found to exhibit structural and morphologic changes of the brain’s mood centers involving the hippocampus (19), amygdala, and prefrontal brain regions (20), which may be affected after concussion. In addition, it pointed out the effect of the apolipoprotein E4 allele (which has been shown to predispose to Alzheimer’s disease) which is associated with chronic traumatic brain injury in boxing (21). That is, in boxers who had been knocked out many times in their boxing career, those with the E4 allele had considerably higher dementia symptoms than others. In other words, the E4 allele influenced whether an individual was relatively sensitive or insensitive to the dementia-causing effects of repeated mild brain injury (22). On the other hand, the psychosocial and cognitive model of depression provided a framework for identifying and understanding factors that maintain an episode of depression (23). In addition, some aspects of self-control and self-efficacy, such as the lack of an active rehabilitation, removal from sport, isolation, and lack of social support in dealing with concussion may influence mood in athletes with concussion (24, 25). Therefore, this study attempts to better understand the factors affecting depression in athletes who engage in individual sports by exploring the influencing factors and mechanisms of depression among boxers. Therefore, investigating the influential factors and mechanisms of boxers’ depression is particularly important to prevent and reduce such depression in boxers and promote their healthy development.

Moving away from medical models that focus on treatment to more preventative and contextual approaches to health care, understanding a broader range of psycho-social outcomes associated with health care prevention is paramount. In this case, that self-control plays a mediating role between self-efficacy and depression in boxers is highly relevant both to social cognitive theory (SCT) and to broader psycho-social considerations of health.

Albert Bandura’s (26, 27) SCT theory represents one of the most studied theories in this field, with applications in contexts as diverse as education, healthcare, and indeed sport and exercise. It is positioned as a theory of human behavior with integrative principles of broad applicability (28). Within SCT, self-efficacy, which represents a person’s beliefs in their capabilities to perform given behaviors (29), is considered to be the focal determinant of task-oriented behavior (30) and drives healthy behavior (27). Previous studies have shown that not only is self-efficacy an important component of sports-success functioning (31, 32), it also has a positive protective effect on athletes’ mental health (33, 34). Therefore, this study is based on SCT, to more clearly demonstrate the importance of self-control and self-efficacy in preventing and reducing depression in boxers.

Extensive studies have found self-efficacy (35), self-control (36), and self-esteem (37) to be important factors affecting depression. Self-efficacy refers to an individual’s belief in his or her organization and ability to perform the necessary actions to achieve a particular goal (38, 39). Research suggest that low self-efficacy leads to depression (29, 40) and that it can be an important protective factor for individual mental health. This kind of hypothesis is confirmed by recent studies, which find self-efficacy to be a significant negative predictor of depression (41–43) and an important factor affecting depression (44, 45). Individuals with high self-efficacy believe that they can effectively control potential external threats, and their positive responses can help them maintain their mental health. However, while some studies have confirmed that self-efficacy would negatively predict depression overall (46–48), no research has specifically examined the relationship between boxers’ self-efficacy and depression. The purpose of the current study was to, therefore, proposed that self-efficacy among boxers would negatively predict depression (Hypothesis 1).

Self-control is an individual’s ability to influence, regulate, and control one’s own psychological, behavioral, and physiological processes (49). Some previous research has found that depression can be seen as a set of related problems in self-control (50). Therefore, self-control can also be used as a strategy to prevent or alleviate depression (51). Research over the past few decades has shown that self-control training can successfully reduce depression levels (52). Previous studies have found self-control to be a key factor affecting depression, and specific research has indicated that it is a negative predictor of the same (53, 54). Studies have shown that individuals with lower levels of self-control are more likely to suffer from depression than those with higher levels (55, 56); the latter is more willing to work at adopting strategies to deal with their problems and have a higher sense of control to fight against pressure. Those lacking the ability to regulate and effectively control their behavior are more likely to be depressed at some point in their life. Thus, it is still unclear whether there is a psychological mechanism affecting self-control and depression among boxers. Based on the above evidence, we hypothesized that self-control would negatively predict depression among boxers (Hypothesis 2).

Self-efficacy signals one’s emotions over self-control; individuals will only act positively if they feel they have the ability to complete an activity (57). Self-control requires an individual to have the resources to control themselves, and self-efficacy is a positive emotion that can complement this ability. Bandura believes in the interaction between self-control and self-efficacy (58). Some studies showed that self-efficacy can positively predict self-control (59–61). However, other researchers have found that, under certain circumstances, individual self-control can significantly negatively predict self-efficacy (62, 63). Thus, there may be different relationships between self-efficacy and self-control among different groups. Therefore, research should explore the influence of boxers’ self-efficacy on self-control.

Many existing studies demonstrated that self-control is closely related to self-efficacy and depression, acting as a mediating variable worth considering, and that it mediated the relationship between other psychological characteristics and depression in different groups (64–66). This study, thus, was to investigate whether self-efficacy has a positive effect on boxers’ self-control and whether there is a mediating effect on the relationship between self-efficacy and depression. This study speculates that, among boxers, there is a positive correlation between self-efficacy and self-control, and that self-control mediates the effects of self-efficacy on depression (Hypothesis 3).

### The Current Study

**Figure 1:**
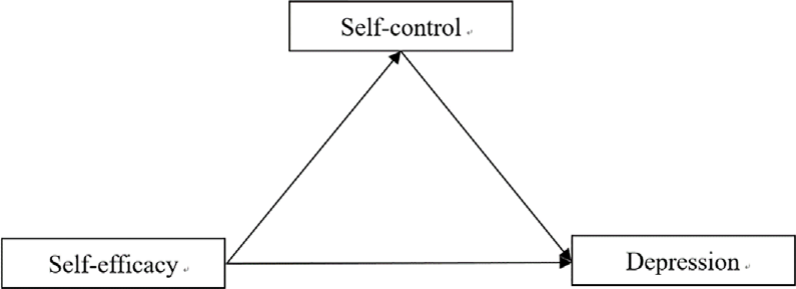
The proposed moderated mediation model.

The aim of the current study was to investigate the effect of boxers’ self-efficacy, self-control, and depression. The hypothesis is that boxers’ self-efficacy would be significantly negatively related to depression, and boxers’ self-efficacy is significantly positively predictive of self-control; secondly, boxers’ self-control would significantly negatively predict depression; finally, boxers’ self-control has a significant mediating effect between self-efficacy and depression. Therefore, this study explored the effect of self-efficacy on the depression of boxers and its internal mechanism from the perspective of the protective mechanism of self-efficacy on individual mental health. How does boxers’ self-efficacy affect depression? (The mediating role of self-control in it). We aimed to provide sport psychologists with new perspectives and helpful suggestions for the intervention and treatment of boxers’ depression. Thus, we simulated a mediation model to examine whether boxers’ self-control mediates the relationship between self-efficacy and depression. **Figure 1** illustrates the conceptual model applied.

## Methods

### Sample

The sample comprised of boxers from China, individuals and teams. This study adopted cluster sampling and selected boxers from China in different cities as participants to complete a survey questionnaire. In a cross-sectional study, a total of 250 questionnaires were distributed, and a total of 231 valid questionnaires were returned, a response rate of 92%. Among the participants, *N = *144 were male (62%), *N = *87 were female (38%). Their average age was *M*= 20.28 years (*SD* = 2.60, ages around 18 to 32).

### Procedure

All participants were invited to complete an anonymous online survey, which took approximately 20 min to complete. Participants were approached by the researchers, with the support of the participating sporting codes, consistent with the approved research ethics procedures. The ﬁrst page of the survey included informed consent information, including the voluntary nature of participation. This ﬁrst page of the survey stated that consent was inferred based on the provision of data. Participants were reminded of any missing items prior to progressing to the next page, resulting in no missing outcome data. Participants were provided with individual access to a tablet or phone to complete the survey. Data collection occurred locally, at each of the participant clubs/teams.

### Measures

#### Self-Efficacy Scale for Athletes

The self-efficacy scale for athletes consists of 15 items (67), such as “I can keep my mind clear and focused during the competition”. Items for the self-eﬃcacy scale ranged from *1= never been like this* to *5=always so*. A confirmatory factor analysis confirmed the one-dimensionality of the scale (CFA): *χ2* = 97.75, *df=*70, *χ2/df *=1.40, RMSEA =0.042, IFI =0.983, NFI =0.943, CFI =0.983. The factor loadings of the items ranged from a =0.44 to a = 0.68. The internal consistency of the questionnaire was good (α = 0.92).

#### The Center for Epidemiologic Studies Depression (CES-D) Scale

The CES-D scale contains 20 items (68). The questionnaire included 20 items; one example was “I feel depressed.” This item had to be answered on a scale of 1 (rarely or none of the time) to 4 (most or all the time), with higher scores indicating more depressive symptoms. A confirmatory factor analysis confirmed the one-dimensionality of the scale (CFA): *χ2* = 3.21, *df=*2, *χ2/df *=1.61, RMSEA =0.051, IFI =0.993, NFI = 0.982, CFI = 0.993. The factor loadings of the items ranged from a =0.35 to a =0.71. The internal consistency of the questionnaire was satisfactory (α = 0.70).

#### Self-Control Questionnaire

Self-control was measured by a 19-item (49), five-point Likert scale questionnaire, ranging from “1 = not at all” to “5 = very much”, which reflects the five higher-order domains of impulsive control, work or study performance, healthy habit, moderation entertainment and resist the temptation; higher scores indicate a better self-control (item example: “I am good at resisting temptation”). A confirmatory factor analysis confirmed the one-dimensionality of the scale (CFA): *χ2* = 6.19, *df=*3, *χ2/df *=2.06, RMSEA =0.068, IFI =0.989, NFI =0.979, CFI =0.989. The factor loadings of the items ranged from a = 0.47 to a =0.78. The internal consistency of the questionnaire was well-qualified (α = 0.83).

### Data Analysis

This study used SPSS 22.0 for statistical analysis and Amos 24.0 for establishing the structural model.

Firstly, we used initial correlational analysis to examine the relationships between self-efficacy, self-control, and depression. Descriptive statistics and means (M) and standard deviations (SD) were tested *via* IBM SPSS Statistics version 22. Then, following the two-step procedure recommended by Gerbing and Anderson (69), this study tested the measurement mediation model before construction (70). We first used a measurement model that contained three potential variables: self-efficacy, self-control, and depression, to test whether each latent variable could be well-represented by its indicators. We next determined whether the results from the measurement model were satisfactory; the structural model could be tested using the maximum likelihood (ML) estimation in the AMOS 24.0 program. To control the inflation of measurement errors generated by multiple items for the latent variable, we created several parcels using the item parceling assignment method (71), and specially created two-item parcels for self-efficacy, five-item parcels for self-control, and four-item parcels for depression.

To assess the adequacy of the model fit, we used the following six goodness-of-fit indices (72): 1) chi-square statistics between 1 and 3; 2) a standardized root mean square residual (SRMR) of 0.06 or less; 3) a root-mean-square error of approximation (RMSEA) of 0.08 or less; 4) a goodness-of-fit index (GFI) of 0.90 or higher; 5) a Tucker-Lewis index (TLI) of 0.90 or higher; and 6) a comparative fit index (CFI) of 0.90 or higher.

## Result

### Preliminary Analysis

Preliminary analyses for the study variables were presented in **Table 1**. Firstly, boxers’ self-efficacy was negatively correlated with depression, and boxers’ self-control negatively correlated with depression. Moreover, boxer’s self-efficacy was positively correlated with self-control. Therefore, in this study, the significant correlations between the variables provided a basis for subsequent testing of mediating effects.

**Table 1:** Descriptive statistics for study variables (*N*=231).

	*M*	*SD*	1	2	3
**1. Self-efficacy**	3.34	0.65	–		
**2. Self-control**	3.44	0.51	0.23***	–	
**3. Depression**	2.10	0.30	-0.21**	-0.41***	–

*indicates P < 0.05, **indicates P < 0.01, and ***indicates P < 0.001.

### Overall Model Analysis

According to the testing procedure of mediating eﬀects (Preacher et al., 2006). Firstly, the ﬁtness indicators of the SEM direct eﬀect analysis results were as follows: (*χ2* = 8, *df*=15.52, *χ* ^2^/*df* = 1.940, *p*< 0.05), CFI (0.983), the TLI (0. 967), and the GFI (0.977)>0.90, and the RMSEA (0.067) <0.06. Self-efficacy was negatively related to depression (*β*=−0.24, *p* < 0.001). Then, the SEM tested whether there was a mediating effect of self-control between self-efficacy and depression in boxers. The chi-square value was signiﬁcant (*χ^2 =^*38, *df*=55.73, *χ* ^2^/*df* =1.467, *p* < 0.05) with its sensitivity to the large sample size, other goodness-of-ﬁt indices demonstrated satisfactory results for this study, with the CFI (0.978), the TLI (0.969), and the GFI (0.957)>0.90, the RMSEA (0.045)<0.06, and the SRMR (0.047) <0.06. The variance in depression explained by this model was 35%. All of the observed variables were signiﬁcantly loaded on the latent constructs in the expected directions, which suggested that the selected indicators reasonably represented the underlying constructs in a statistically reliable manner.

**Figure 2:**
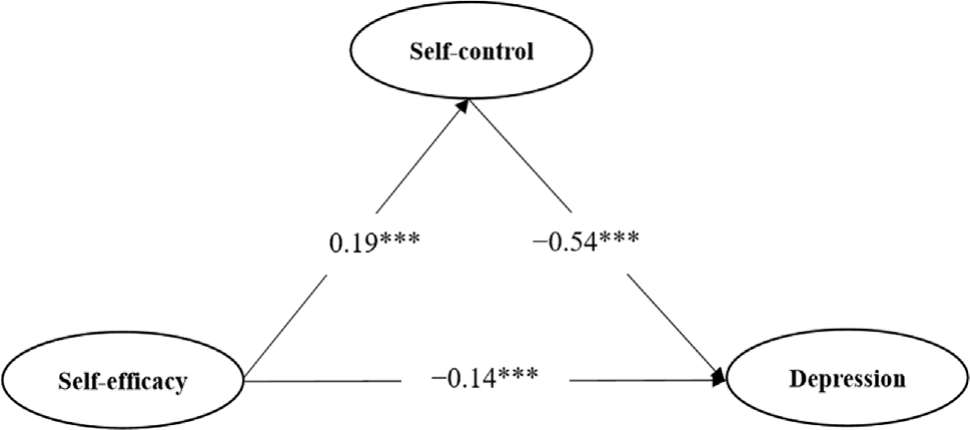
Structural equation modeling of direct and mediating eﬀects on boxers’ depression. ***indicates P < 0.001 and ***Statistically significant.

As shown in **Figure 2**, self-efficacy was negatively related to depression (*β*=−0.14, *p* < 0.001), and self-control was negatively related to depression (*β*=−0.54, *p* < 0.001). Moreover, self-efficacy was positively related to self-control (*β*=0.35, *p* < 0.001), and age was positively related to self-control (*β*=0.19, *p* < 0.001). Taken together, self-control had a partial mediating effect on the relationship between self-efficacy and depression.

## Discussion

This study discussed the relationship between boxers’ self-efficacy and depression and its internal mechanisms of action. Correlation analysis stated that there is a significant negative correlation between boxers’ self-efficacy, self-control, and depression, and there is a significant positive correlation between self-efficacy and self-control. In addition, boxers’ self-control has a significant mediating effect between self-efficacy and depression.

This study aims to understand whether self-efficacy and self-control can predict depression among boxers. We determined self-efficacy to be signiﬁcantly and negatively associated with depression, a finding that supports Hypothesis 1 and is consistent with previous ﬁndings (41–43). Our results also suggested that boxers’ self-efficacy would negatively predict depression; again, this finding was consistent with those of previous studies (45–48). Social cognitive theory argues that a lack of self-efficacy might lead to feelings of depression through a discrepancy in aspirations and perceived skills (73). The results of this study to a certain extent support the views of social cognitive theory, that self-eﬃcacy inﬂuences depression in boxers. So, self-efficacy is so important for boxers, it is a psychological indicator to predict the depression of boxers. In addition, some studies that rely on longitudinal study methods also show a significant negative correlation between self-efficacy and depression (44, 74), while others demonstrate that reduced levels of individual self-efficacy lead to psychological maladaptation, including depression (75, 76). Thus, improving boxers’ self-efficacy could not only enhance the probability of winning competitions, but also contribute to boxers’ mental health.

Our results also suggested that self-control is signiﬁcantly and negatively associated with depression among boxers, we found that this supports Hypothesis 2 and is in accordance with prior studies (53, 54). A self-control model of depression found that self-control therapy showed a significantly greater reduction in depression (26). The results of this study to a certain extent support the views of a self-control model of depression, that improving boxers’ self-control helps reduce their risk of depression. High levels of self-control are vital for the healthy development of an individual’s physical and mental health (49). In addition, a large number of studies have found that people who report depressive symptoms are more likely to be impulsive and have a lower level of self-control than those without such symptoms (77–79). In sporting contexts, self-control is a key factor influencing athletic performance (80). Overall, for boxers, improving self-control levels not only helps improve athletic performance but also has a positive effect on reducing the risk of depression. Conversely, if boxers have a low level of self-control, their risk of depression increases.

Finally, this study found that boxers’ self-control plays a significant mediating role between self-efficacy and depression; this finding supports Hypothesis 3 and is in line with previous research (64–66). Bandura’s (81) model of self-control with the addition of attributional considerations is proposed as a heuristic model for the analysis of the phenomena of depression. The results of this study support this model. The results show that boxers’ self-control plays a partial mediating role in the relationship between self-efficacy and depression. Boxers’ self-efficacy not only has a direct impact on depression, but also indirectly through self-control. Therefore, no matter where boxers are in competition and life, if the level of self-efficacy and self-control is high, the risk of depression is reduced. Thus, future research should focus on improving boxers’ self-efficacy and self-control to prevent depression during high-pressure training or competitions.

In summary, the results of this study are not only applicable to boxers but can also be promoted among other athletes. This new conceptual framework can be a valuable and novel perspective for the future research of depression in applied ﬁelds such as sport, providing possible targets for intervention, and forming a basis for further research. In addition, boxing is a confrontation sport that increases physical fitness and is loved by different age groups for the role the sport can play in helping to raise awareness of mental health generally.

## Conclusion

To sum up, we used the self-efficacy scale for athletes, the self-control questionnaire, and the Center for Epidemiologic Studies depression scale to collect data from 231 Chinese national boxers. Based on SCT, this study clearly demonstrated the importance of self-control and self-efficacy in preventing and reducing depression in boxers. Therefore, for the mental health education of boxers, on the one hand, it is necessary to continuously improve their self-control levels, and at the same time, improve their self-efficacy through behavioral training which will be more conducive to reducing the depression of boxers. Future studies could replay the same experimental procedures in these contexts to provide more substantial findings and consider using specific questionnaires on personality profiles.

## Limitations

This research has certain theoretical and practical significance, but there are some limitations. Firstly, this study uses a cross-sectional study design, making it difficult to make accurate causal inferences. On the other hand, the survey instruments have not been validated for use in boxers. The findings of this study can be tested in the future through experimental research and tracking design. Secondly, only the mediating effect of self-control between boxers’ self-efficacy and depression is considered, but in reality, there are still other mediator variables, such as self-esteem and personality, which are subject to further research.

## Data Availability Statement

The datasets generated for this study are available on request to the corresponding authors.

## Ethics Statement

The study was approved by the Research Ethics Committee of Southwest University, Chongqing, China. Written, informed consent was obtained from all the participants.

## Author Contributions

XC, NQ, CC, DW, GZ, and LZ conceived the study, interpreted the data, drafted and revised the work, approved the final version of the manuscript to be published, and agreed to be accountable for all aspects of the work.

## Funding

This work was partly supported by the China Scholarship Council (No. 202008080341).

## Conflict of Interest

The authors declare that the research was conducted in the absence of any commercial or financial relationships that could be construed as a potential conflict of interest.
